# Supplementary material for: Ion complexation waves emerge at the curved interfaces of layered minerals
Source: Nat Commun. 2022 Jun 13;13:3382. doi: 10.1038/s41467-022-31004-0 (PMC9192655; doi:10.1038/s41467-022-31004-0)
Supplement: Supplementary file 1 — Supplementary Information [file 41467_2022_31004_MOESM1_ESM.pdf]

Supplementary Materials for

Ion complexation waves emerge at the curved interfaces of layered minerals

Michael L. Whittaker, David Ren, Colin Ophus, Yugang Zhang, Benjamin Gilbert, Laura Waller, Jillian F. Banfield
Correspondence to: mwhittaker@lbl.gov

This PDF file includes:

Supplementary Text
Figs. S1 to S18

***1. Suspension dynamics*......2**
1.1. Static X-ray scattering......2
1.2. XPCS......4
***2. Suspension microstructures*......10**
2.1. cryoET......10
***3. Complexation wave model*......14**

1. Suspension dynamics

1.1. Static X-ray scattering

Clay mineral microstructures are often inferred from their average interlayer distances, <*D*>, which in the simplest case of a nematic phase with planar and parallel layers, can be determined from

$$\langle D\rangle = t\phi^{-1} \quad (1)$$

Equation (1) represents the maximum average interlayer spacing for a given mineral volume fraction, ϕ, and is valid above $\phi = 2\%$ for Li-Mt and approximately valid below it (Fig. S1).

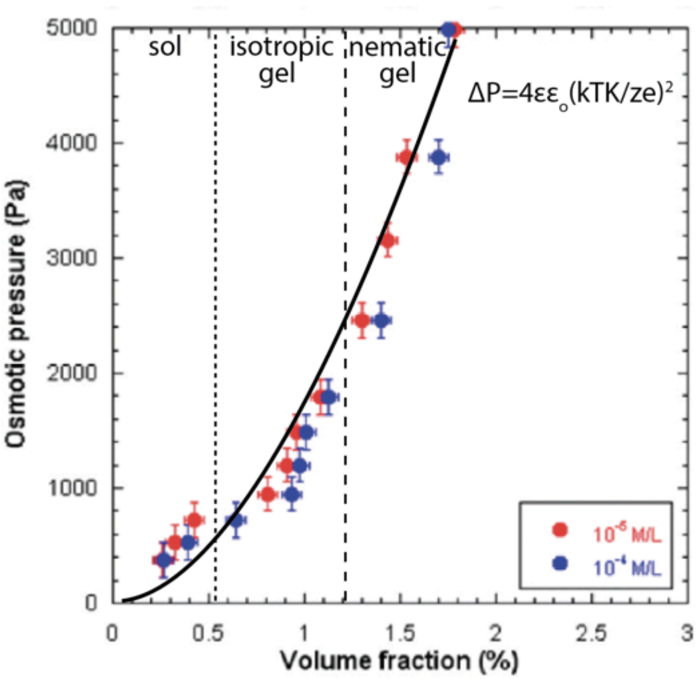

Figure S1. Osmotic pressure of Na-Swy-2 as a function of mineral volume fraction, modified from(*3*) with fit to Eq 4.

The value of <*D*> is typically found from the structure factor, Iq^2, in suspension determined by small-angle X-ray scattering. These values of *D* can be quantitatively reproduced with the osmotic potential from DLVO theory

$$W_{osmotic} = 128C\kappa^{-1}tanh\left(\frac{ze\psi}{4kT}\right)^2 \quad (2)$$

Where κ^{-1} is the characteristic (Debye) length over which the interface potential decays due to screening by background electrolyte, given by

$$\kappa^{-1} = \left(\sum_i \frac{c_i e^2 z_i^2}{kT\epsilon\epsilon_o}\right)^{-1/2} \quad (3)$$

where c_i and z_i are the concentration and charge of electrolyte species i, e is the fundamental charge, k is the Boltzmann constant, T the absolute temperature, ε is the relative dielectric permittivity and ε_o is the permittivity of free space.

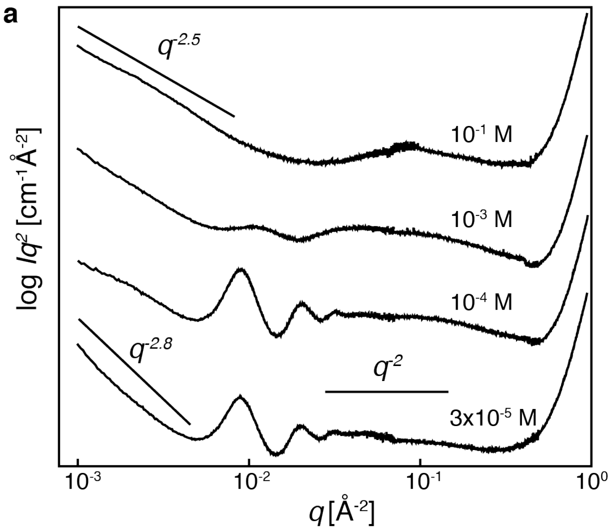

Figure S2. X-ray scattering structure factors from Li-Mt at various electrolyte concentrations. At low background electrolyte the structure factor exhibits oscillations consistent with a nematic phase, while above (10^{-4} M) the structure factor is less ordered and consistent with an isotropic phase.

In the absence of background electrolyte, i.e., the nematic, the osmotic pressure is given by

$$P_{osmotic} = kTC_o \quad (4)$$

where the concentration at the midplane between layers, C_o is determined by the contact value theorem(*4*).

1.2. XPCS

Suspension dynamics determined by XPCS varied widely between Li-Mt, Na-Mt and K-Mt. Correlation between coherent scattering intensity at q = 0.0025 Å^{-1}, corresponding to the approximately 250 nm average diameter of an Mt layer, is shown in Fig. S5. At equivalent mineral (2%) and alkali chloride electrolyte (1 M) concentrations, decreasing cation hydration energy[2] was directly correlated with faster dynamics, which manifest as transitions between correlated (1.18) and uncorrelated (1.00) states.

Equilibrium fluctuations between two-dimensional speckle patterns from coherent X-ray scattering were quantified using the (one-time) intensity autocorrelation function, $g_2(q, t)$, defined at each pixel as

$$g_2(q,t) = \frac{\langle I(q,t)I(q,t+\tau)\rangle_T}{\langle I(q,\tau)\rangle_T^2} \quad (5)$$

where $I(q, t+\tau)$ is the intensity at scattering vector q and time $t+\tau$ for a given timepoint t and T is the duration of acquisition. Values of $g_2(q, t)$ are averaged over a range of q values corresponding to a length scale of interest. For the beamline optics used in this study, 1.18 is the maximum value of $g_2(q, t)$ and a minimum value of 1.0 corresponds to completely uncorrelated structures.
The two-time correlation function, $\chi(q, t_1, t_2)$, is analogously used to define the intensity autocorrelation when the relaxation time constant changes over time. In this non-equilibrium case

$$\chi(q,t_1,t_2) = \frac{I(q,t_1)I(q,t_2)}{\langle I(q,t)\rangle^2} \quad (6)$$

The change in $g_2(q, t)$ with time determined from both one- and two-time intensity autocorrelation functions was fit with a stretched exponential of the form

$$g_2(q,\tau) = 1 + \beta(q)exp(-2D(q)q^2\tau) \quad (7)$$

where $\beta(q)$ is the q-dependent contrast magnitude and $D(q)$ is the q-dependent diffusion coefficient. Diffusion coefficients, Do, were calculated from Equation (7) and found to be highly dependent on the counterion identity. Diffusion coefficients of K-Mt were nearly three orders of magnitude larger than Na-Mt and six orders of magnitude larger than Li-Mt.

We designed our experiments to be conducted with nine different total X-ray doses for each sample condition using three different dose rates. We used an attenuator placed in the beam path prior to interacting with the sample to change the total X-ray dose (4%, 19% and 100% transmission) for a fixed exposure time. We also varied the exposure time among up to three values (1ms, 10 ms, and 100ms) for a given attenuator thickness, producing a total of between six and nine measurements per sample condition.

These results make obvious the fact that the X-ray beam is not driving dynamics. So obvious, in fact, that we deeply regret not providing this full analysis to the Reviewer sooner. We discuss the reasons for this in Part 3. However, the topline conclusion is presented in Fig. 1: the X-ray dose has almost no effect on the observed dynamics, while the solution conditions that we prepared to test our hypothesis have an incredibly large effect (note the log scale on the ordinate axis). Fig. 1 leaves no ambiguity whatsoever about whether X-ray dose is contributing to the effects we observed. In fact, Fig. 1 indicates that we could not produce diffusivities as high as we

observed in the presence of different ions if we instead attempted to do this solely using the X-ray beam, no matter how hard we tried.

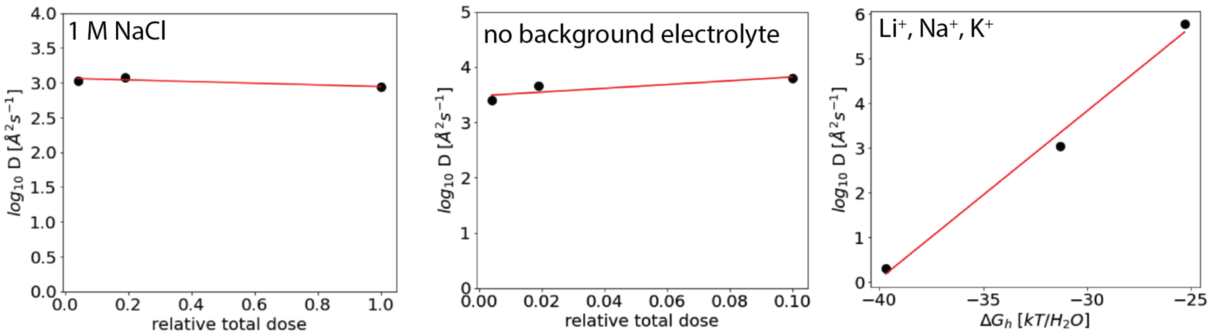

Figure S3. | Comparison of the average diffusion constant, D, of clay layers under different conditions. Total X-ray dose has almost no effect in either 1 M NaCl (left) or in the absence of background electrolyte (center) while the hydration energy of the cation in systems with 1 M chloride salts has a very large effect (right, note logarithmic ordinate scale).

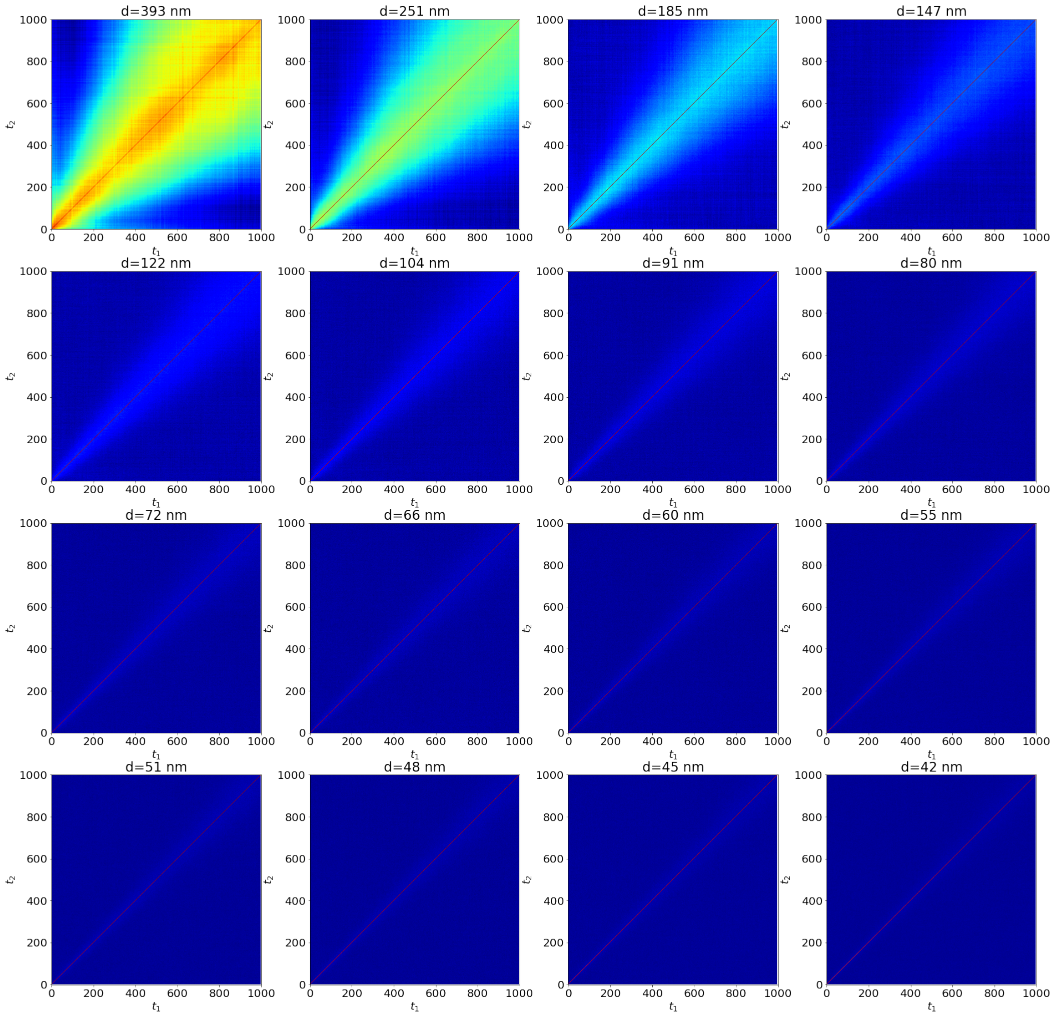

Figure S4. Two-time correlation plots for Na-Mt in water from scattering vectors corresponding to 42-393 nm.

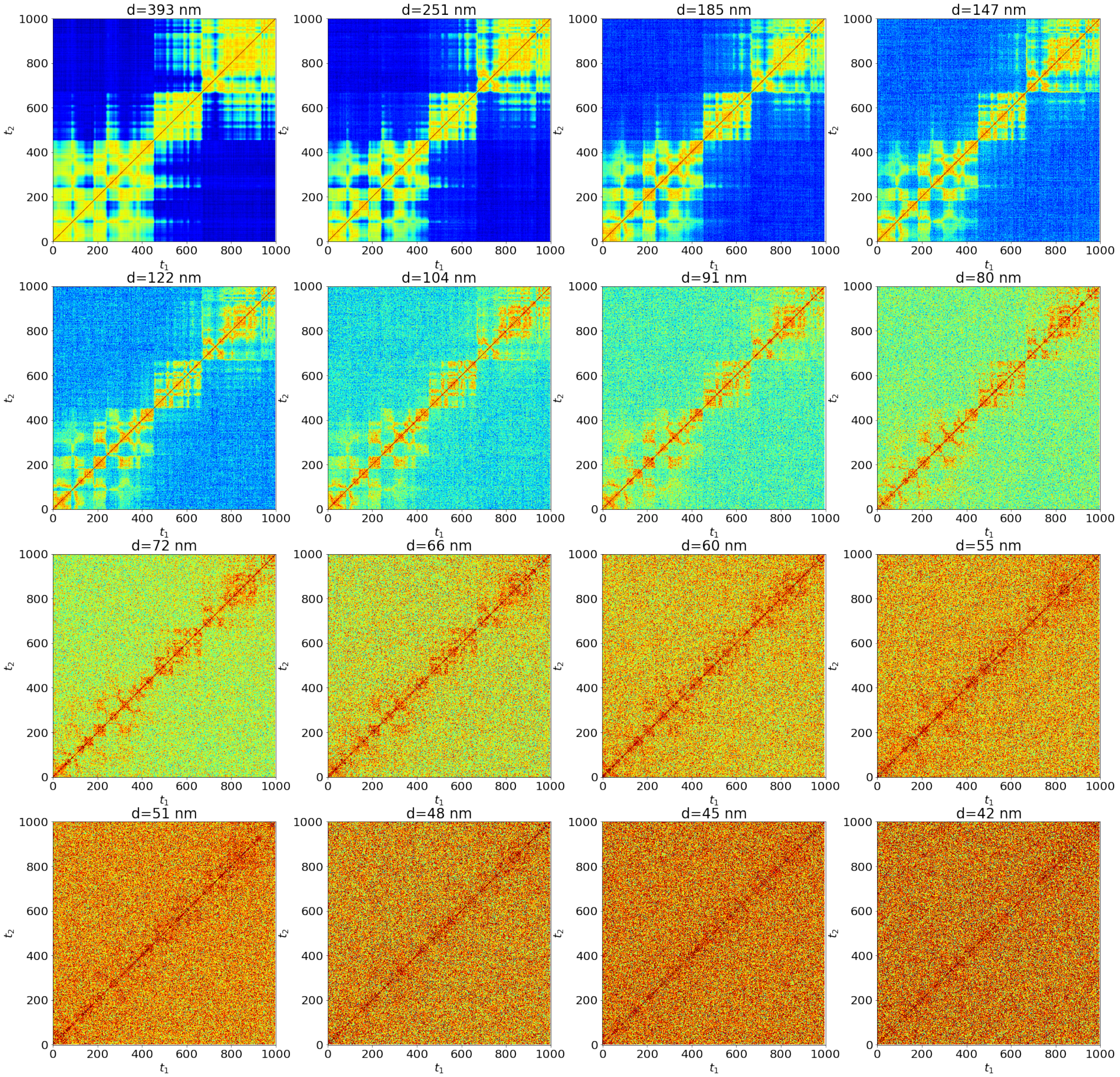

Figure S5. Two-time correlation plots for Na-Mt in 1 M NaCl from scattering vectors corresponding to 42-393 nm.

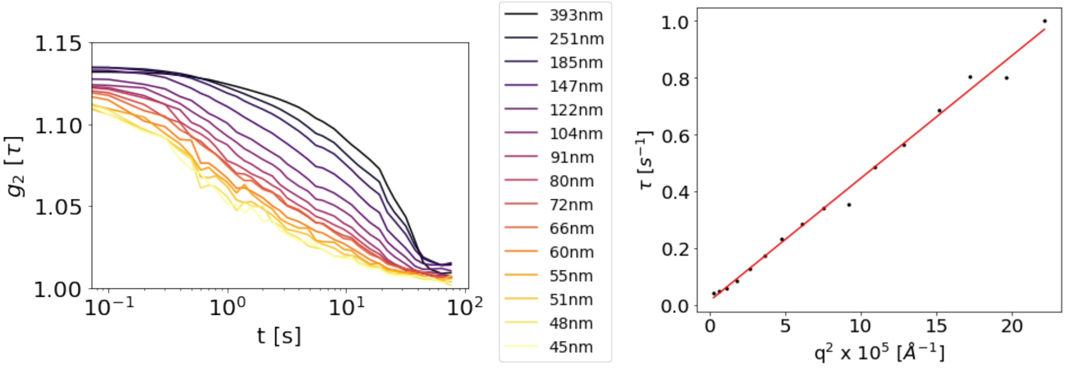

Figure S6. g_2 data corresponding to scattering vectors shown in Fig. S6 for NaMt, showing strong dependence of the relaxation time on the scattering vector across all scattering vectors measured.

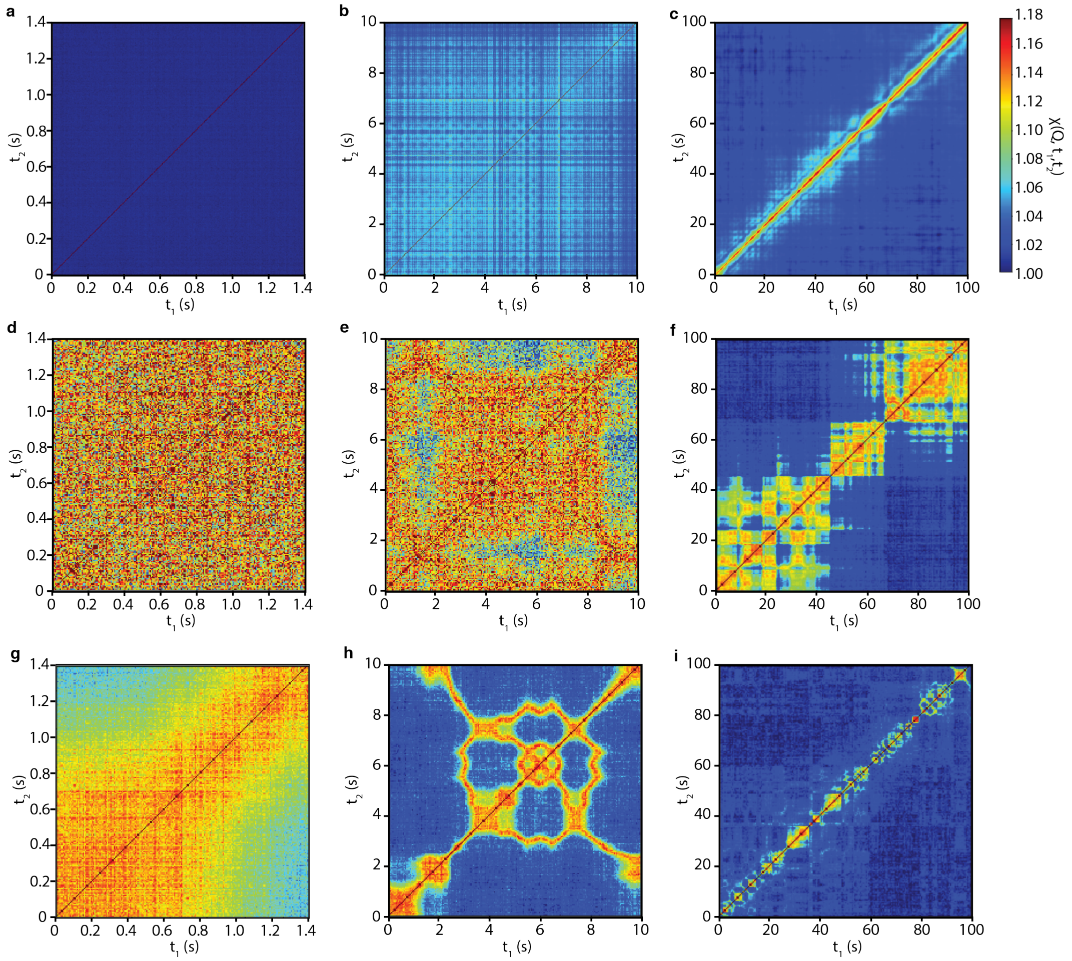

Figure S7. XPCS two-time correlation plots. (a-c) Li-Mt, (d-f) Na-Mt, (g-i) K-Mt.

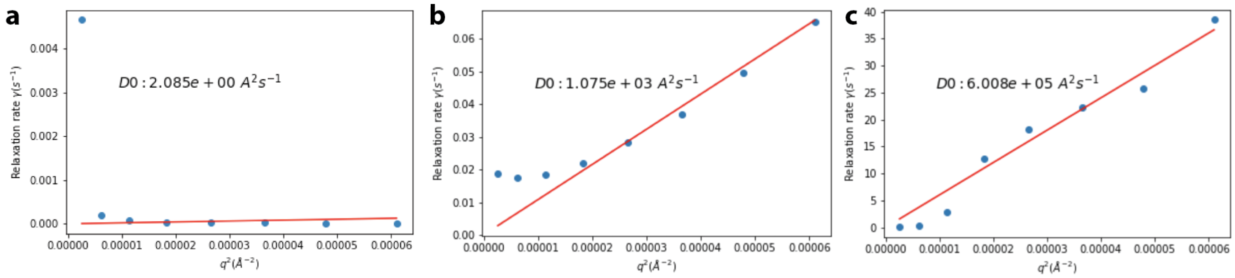

Figure S8. Diffusion coefficients calculated from the size-dependent relaxation time. (a) Li-Mt, (b) Na-Mt, (c) K-Mt.

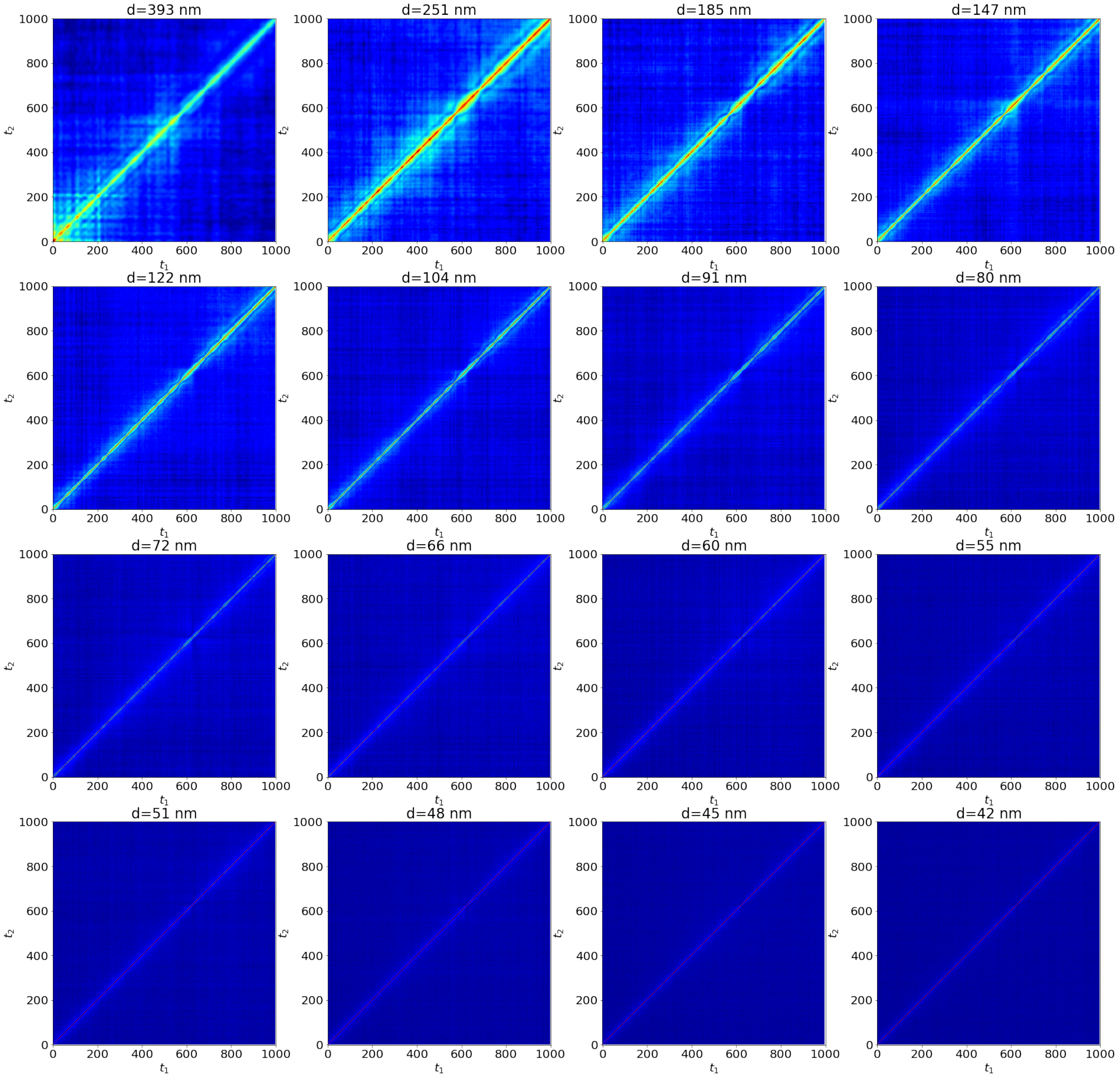

Figure S9. Two-time correlation plots for Li-Mt in 1 M LiCl from scattering vectors corresponding to 42-393 nm.

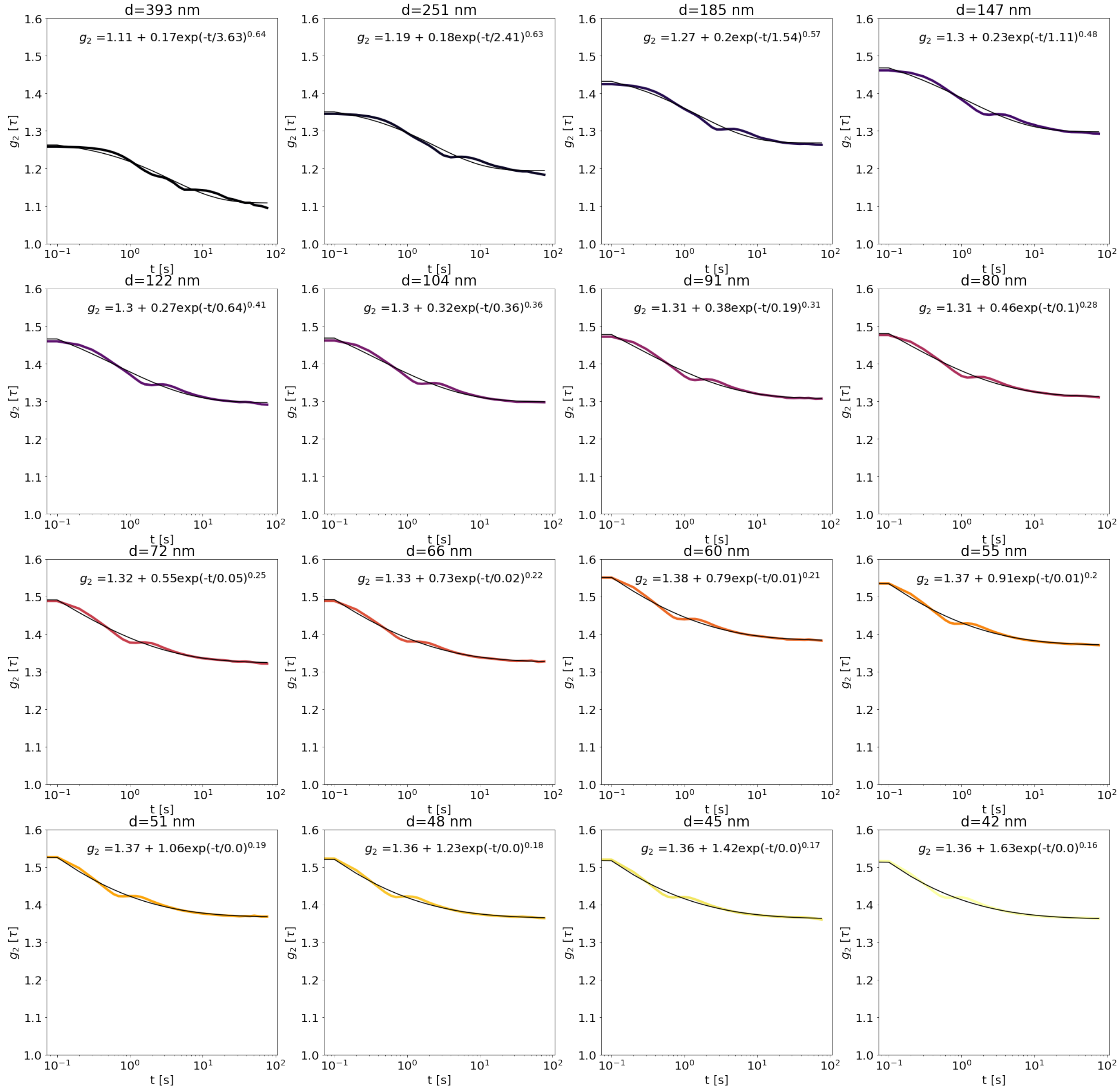

Figure S10. g_2 data and fit functions corresponding to scattering vectors shown in Fig. S9 for Li-Mt, showing strong dependence of the relaxation time on the scattering vector.

2. Suspension microstructures

2.1. cryoET

The clay sheets in the reconstructed cryoET absorption volumes were segmented using custom codes written in Matlab. The below variables were defined for a bin 2 reconstruction size. The goal was to reduce the set of voxels to a set of "sheets" which were defined as a cloud of points representing a 2D sheet embedded in 3D space, each with an associated normal vector representing the sheet surface normal. First, we generated a list of unit vectors $\boldsymbol{n_i}$ with roughly even angular spacing over a hemispherical surface, representing the possible sheet orientations. Next, for each orientation we generated a 3D kernel $\boldsymbol{k_i}$ defined by the function

$$\boldsymbol{k_i} = \frac{1}{2} - \frac{1}{2}\operatorname{erf}\left(\frac{t/2 - |\boldsymbol{r} \cdot \boldsymbol{n_i}|}{w}\right) \tag{8}$$

where $\boldsymbol{r}$ is the real space coordinates centered on the origin, $t = 3$ is the estimated sheet thickness, and $w = 1$ is the estimated sheet interfacial width. This kernel was normalized by applying a 3D Gaussian envelope function and subtracting the mean, i.e. the formula

$$\boldsymbol{k_i}^{\text{norm}} = \left[\boldsymbol{k_i} - \left\langle \boldsymbol{k_i} \exp\left(-\frac{|\boldsymbol{r}|^2}{2\sigma^2}\right)\right\rangle\right] \exp\left(-\frac{|\boldsymbol{r}|^2}{2\sigma^2}\right) \tag{9}$$

where $\sigma = 8$ is the Gaussian envelope standard deviation, and $\langle \quad \rangle$ represents the mean or expectation value. For each potential orientation, we use Fast Fourier Transforms to efficiently compute the correlation of the kernel with the reconstructed volume. We then take the maximum correlation value in each voxel over all orientations, while also storing the best-match orientation. We then classified the sheet voxels by applying two thresholds: (1) a global threshold by using a minimum value for the correlation signal, and (2) voxels with correlation signals greater than at least 18 neighboring voxels (out of a possible 26 neighbors).

Next, we computed the nearest neighbor network for the set of all sheet voxels. This network was used to segment the set into separate sheets using two matching rules: (1) locally connected voxels, and (2) those with orientations within 30° of each other. At this stage we also discarded sheets which consisted of less than 1000 voxels, since these were either false positives or sheets too small to make accurate measurements of the surface topology.

Finally, we refined the sheet voxel coordinates and surface normal using an iterative procedure. The first step consisted of moving a voxel to the center position of a parabolic surface fitted to all of the neighboring sheet voxels within 55 voxels. Next, the sheet orientation was updated to be the normal of this parabolic surface at the fitted voxel position. Finally, the voxel was moved perpendicular to the surface (along the orientation normal vector) to the region of lowest absorption signal in the original reconstructed volume. This process was used to remove the "pixelization" of the originally detected coordinate positions. We repeated these steps until all points had converged.

The final analysis steps consisted of measurements performed on the segmented surfaces. Line traces were defined using the surface normal vectors at each point. Mean surface curvature $2H$ was estimated by fitting the 2D parabolic local surface to the expression

$$2H = \frac{\left(1+{S_x}^2\right)S_{yy} - 2S_xS_yS_{xy} + \left(1+{S_y}^2\right)S_{xx}}{\left(1+{S_x}^2+{S_y}^2\right)^{3/2}} \qquad (10)$$

Where S_x and S_y are the first order spatial derivatives of the surface, and S_{xx}, S_{yy}, and S_{xy} are the second order derivatives.

The large field of view in cryoET enables comparison to structural information accessible by *in situ* X-ray scattering of equivalent samples over two orders of spatial magnitude. We observe that the average interlayer spacing, $\langle D \rangle$, reflected by a peak in the reciprocal-space structure factor that is commonly used to characterize the structures and interaction forces in layered mineral suspensions(*5*), differs between electron- and X-ray-based techniques (Fig. 1c, f). This indicates that the structure sampled by cryoET, with a volume of 0.29 μm^3, differs from the ensemble structure of a suspension within the approximately 10^8 μm^3 volume sampled by the X-ray beam. Therefore, X-ray profiles are not adequately representative of individual suspension microstates from which interaction forces arise. In other words, the X-ray structure factor cannot be uniquely inverted to produce the irregular distribution of layer spacings that is obviously present in Fig. 1a. Only from cryoET can we determine that positionally ordered regions like that observed in Fig. 1b exist in some places but not others, and that not all layers participate in such ordered stacks.

The use of normal vectors to extract ion profiles in Fig. 4d-f leads to reproducible trends in ion distributions, despite capturing broad distributions of interfacial curvature and interlayer distances. Often, profiles do not converge at large distances because of the presence of neighboring layers. Such a case is presented in Fig. 4c, in which the neighboring layers are shown in gray. The interlayer distance is not uniform between neighboring layers, and profiles taken normal to the midplane of a layer (over which the degree of curvature, and thus the orientation of the normal vector, vary considerably) extend over a large range of distances before encountering a neighboring layer (and the interfacial ion distribution associated with that layer).

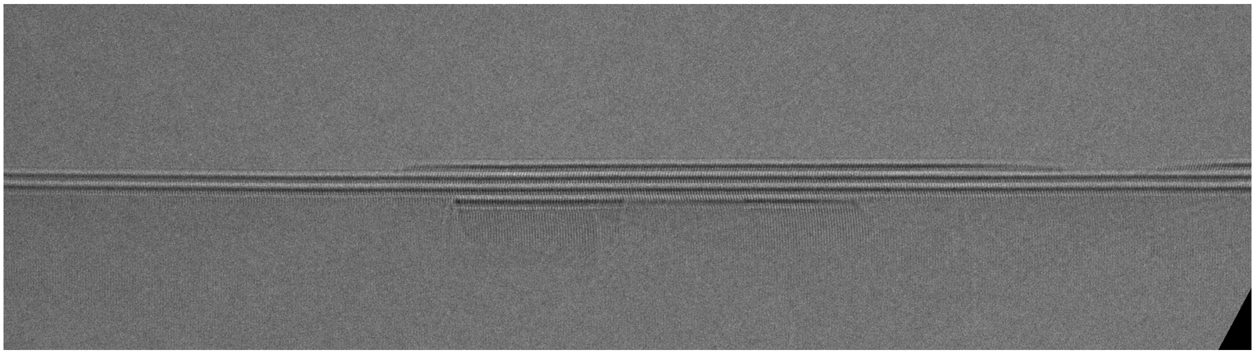

Figure S11. CryoEM image of Na-Mt stack viewed edge-on in Fig. 3. Lattice fringes observed on bottom side of layer are absent on top, indicating that all layers in the stack are curved with concavity facing downward.

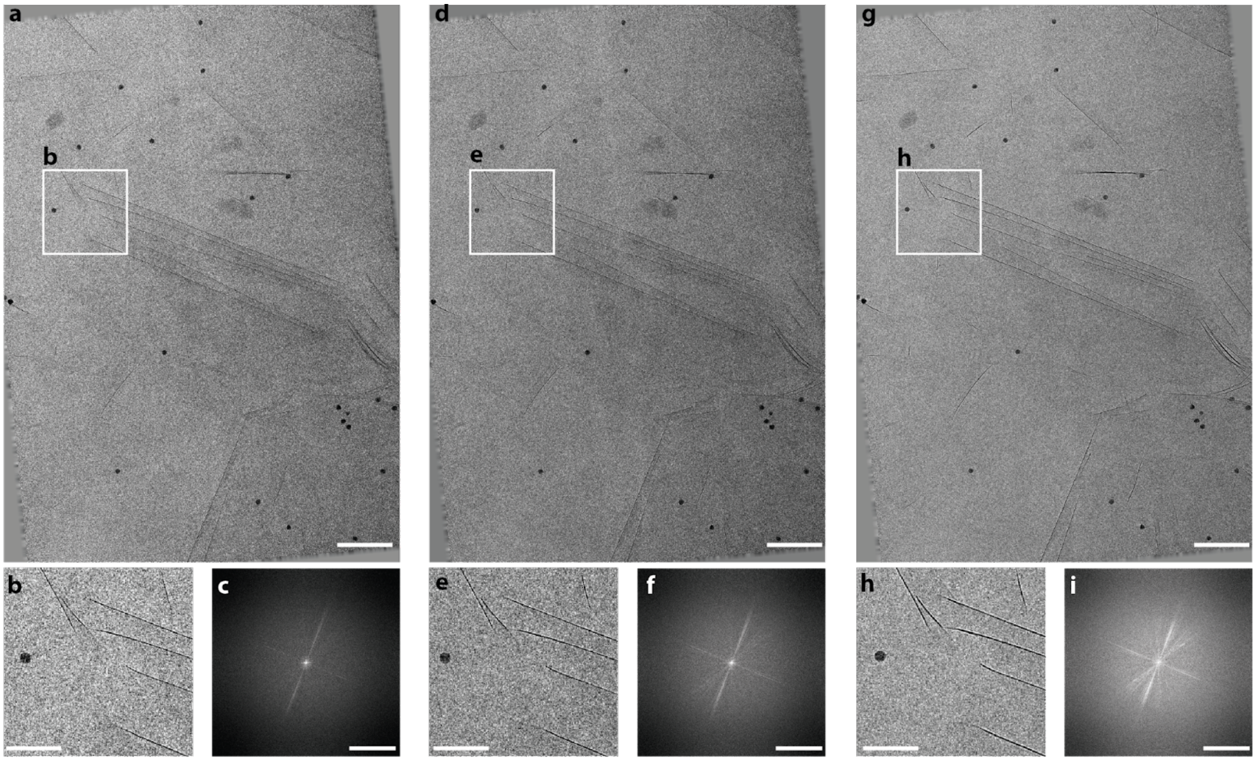

Figure S12. Images taken with different defocus values at the same tilt angle as part of the tilt series from which the tomogram in Fig. 1a was reconstructed. Near 0 nm defocus (a-c) the contrast is weakest (a-b) but the information transfer is highest (c). At -550 nm defocus (g-i) the contrast is highest (g-h) but the information transfer is reduced (i). Scale bars are 100 nm in (a, d, g), 50 nm in (b, e, h) and 1.2 Å^{-1} (c, f, i).

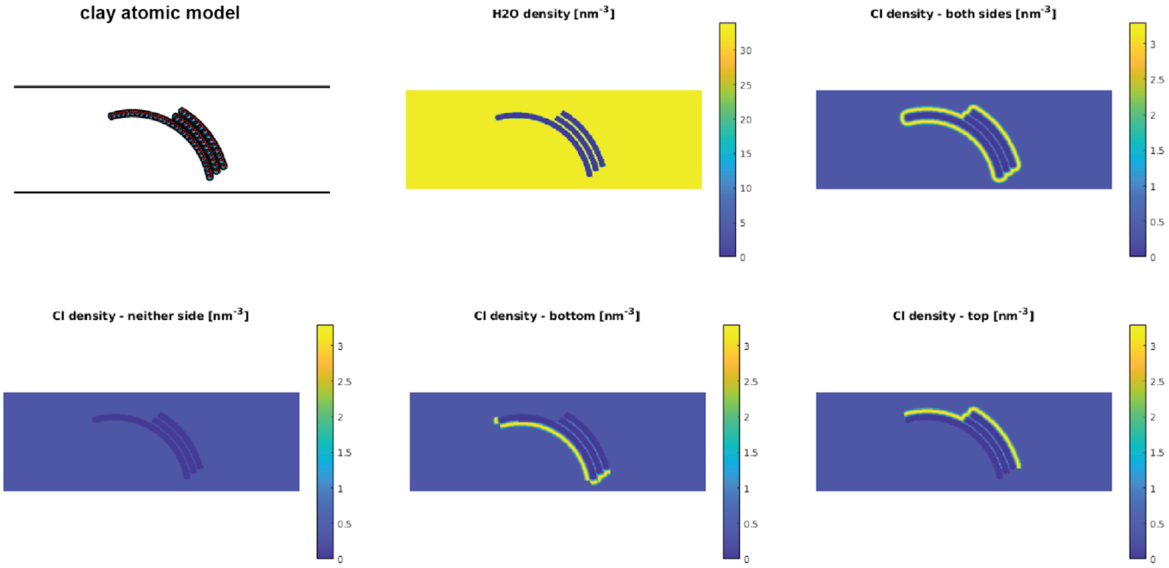

Figure S13. Clay stack and ion configurations used for image and tomogram simulation.

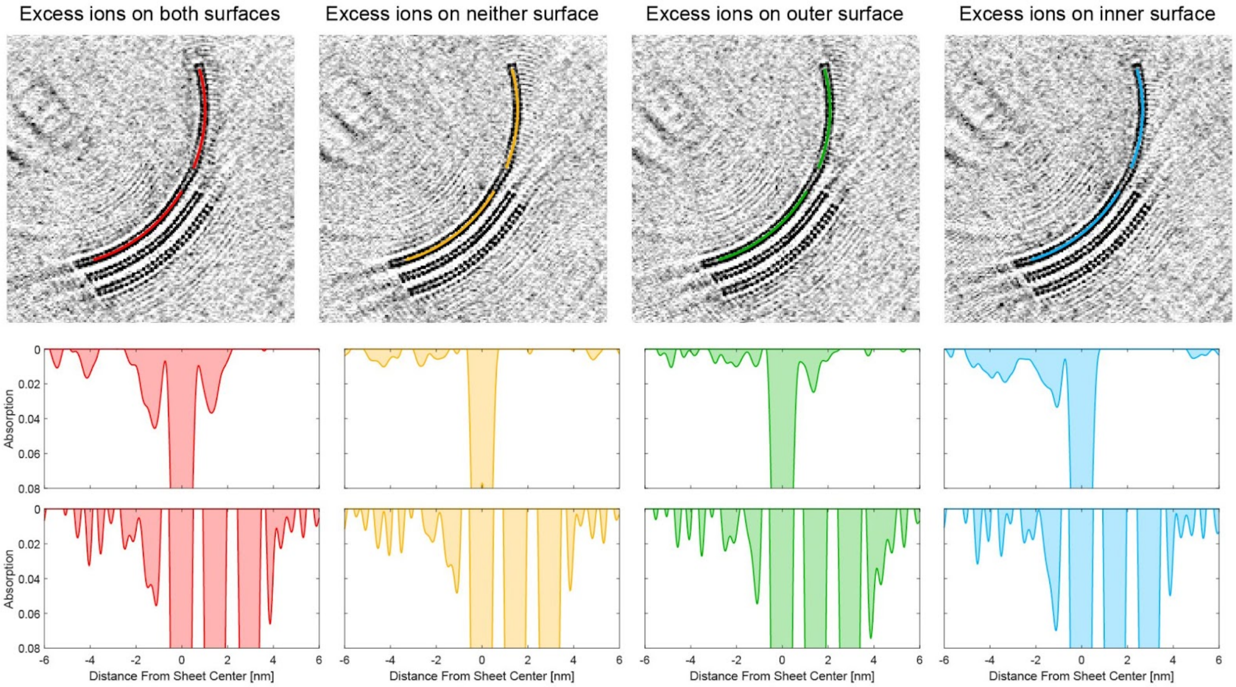

Figure S14. Integrated ion density profiles in tomographically reconstructed simulated images from structures shown in Fig. S2. Differences in ion densities can be observed between the four cases in 2D slices of 3D reconstructions (top row). These differences are clear in 1D profiles adjacent to the single isolated layer (middle row) and are also present in the stacked region despite the presence of artifacts due to the small reconstruction volume.

3. Complexation wave model

We model ion complexation using a Langmuir-type model with two cation configurations. A fraction, α, of the structural charge sites are compensated by hydrated, outer sphere cations. Cations form inner-sphere, partially-dehydrated complexes with a fraction 1-α. There are two sets of complexation sites per unit of structural charge, one on either side of the layer. Thus, each side of a charge-neutral layer has half of all charge sites occupied by a cation in either inner- or outer-sphere configurations, while half of the sites remain unoccupied.

An even balance of inner-and outer-sphere complexes is not strictly true in general, e.g., for curved layers, because there is a near-field contribution from the interaction between structural charges within the layer and those complexed at the surface that augments the far-field potential drop expected from classical EDL theories. This near-field contribution can be modeled using an equivalent circuit, treating the Stern layers and the layer itself as capacitors in series, and the inner- and outer-sphere sites as parallel contributions with distinct dielectric constants.

A general description of complexation equilibria include the transition from bulk solution to an outer-sphere position

$$X^{+}{}_{(aq)} \leftrightarrow X^{+}{}_{(outer)} \tag{11}$$

This reaction has an equilibrium constant

$$K_{outer} = \frac{[X^{+}{}_{(outer)}]}{[X^{+}{}_{(aq)}]} = e^{-\frac{\Delta G^{o}_{c,outer}}{kT}} \tag{12}$$

where $\Delta G^{o}_{c,outer}$ is the standard free energy for outer-sphere complexation. Equilibrium between Outer and inner spheres is given by

$$X^{+}{}_{(outer)} \cdot H_2O + Mt^{-} \leftrightarrow MtX_{(inner)} + H_2O \tag{13}$$

The equilibrium constant for this reaction is

$$K_{inner} = e^{-\frac{\Delta G^{o}_{c,inner}}{kT}} = \frac{[MtX_{(inner)}](a_{H_2O})}{[X^{+}{}_{(outer)}][Mt^{-}]} \tag{14}$$

where $\Delta G^{o}_{c,inner}$ is the standard inner-sphere complexation free energy. Combining Equations (12) and (14) gives

$$K_{inner} = e^{-\frac{\Delta G^{o}_{c,inner}}{kT}} = \frac{[MtX_{(inner)}](a_{H_2O})}{e^{-\frac{\Delta G^{o}_{c,outer}}{kT}}[X^{+}{}_{(aq)}][Mt^{-}]} \tag{15}$$

$$e^{-\frac{\left(\Delta G^{o}_{c,inner}+\Delta G^{o}_{c,outer}\right)}{kT}} = e^{-\frac{\Delta G^{o}_{c}}{kT}} = \frac{[MtX_{(inner)}](a_{H_2O})}{[X^{+}{}_{(aq)}][Mt^{-}]} \tag{16}$$

where ΔG_c^o is the standard complexation free energy difference between an electrolyte counterion in the bulk solution and one forming an inner-sphere complex with the mineral. The fraction of outer-sphere cations, α, is given by

$$\alpha = \frac{[Mt^-]}{[MtX_{Stern}]+[Mt^-]} \tag{17}$$

The concentration of inner-sphere cations can be calculated from Equation (16)

$$\left[MtX_{(inner)}\right] = \frac{[X^+{}_{(aq)}][Mt^-]}{(a_{H_2O})} e^{-\frac{\Delta G_c^o}{kT}} \tag{18}$$

which when combined with Equation (17) gives an alternate expression for α

$$\alpha = \frac{1}{1+\left[\frac{[X^+{}_{(aq)}]}{(a_{H_2O})} e^{-\frac{\Delta G_c^o}{kT}}\right]} \tag{19}$$

Rearranging Equation (19) gives an expression for α in terms of the complexation free energy

$$\Delta G_c^o = -kTln\left(\frac{1-\alpha}{\alpha}\frac{a_{H_2O}}{[X^+{}_{(aq)}]}\right) \tag{20}$$

Global charge balance requires that α = 0.5 at standard state. The reason for this derives straightforwardly from the condition of charge neutrality. The interfacial charge must go to zero in the collapsed, anhydrous state and this is accomplished by inner-sphere complexation of half of the counterions to each side of a layer. Therefore, the standard complexation free energy is

$$\Delta G_c^o = -kTln\left(\frac{1-\alpha}{\alpha}\right) = 0 \tag{21}$$

Equation (21) demonstrates that the standard state is defined relative to an electrically neutral - and as will become apparent, planar - layer. At standard state $\left[X^+{}_{(aq)}\right] = 1M$, $a_{H_2O} = 1$ and $a_{mineral} = 1$. However, standard state cannot be physically realized when the system is defined to include water, electrolyte, and mineral, because the mole fractions are always < 1 and therefore the component activities must vary.

3.1. Generalized surface complexation

In the general case of a curved, charged interface, counterions are distributed between the inner and outer sphere complexation configurations and the total complexation free energy is

$$\Delta G_c = \Delta G_{c,inner} + \Delta G_{c,outer} + \Delta G_{c,bend} + \Delta G_{c,mix} \tag{22}$$

3.2. Bending

The bending free energy at constant hydrostatic pressure is given by(*6*)

$$\Delta G_{c,bend} = \int \left[\frac{k_1}{2}(H_1 + H_2 - H_o)^2 + k_2\left(\frac{1}{H_1 H_2}\right)\right] dS \qquad (23)$$

Where H_1 and H_2 are the mean and Gaussian curvature, k_1 and k_2 are the mean and Gaussian bending moduli, and the integral is over the surface. For a Mt layer with no spontaneous curvature and that exhibits cylindrical symmetry there is only a single radius of curvature and

$$\Delta G_{c,bend} = \frac{1}{2} k_1 (H_1)^2 \qquad (24)$$

Total bending energy includes contributions from both the EDL and elastic strain in the layer

$$k_1 = k_{1,el} + k_{1,EDL} = \frac{Et^3}{12(1-\nu^2)} + \frac{3\pi\sigma^2}{2\varepsilon\varepsilon_o\kappa^3} \qquad (25)$$

3.3. Outer sphere

The outer sphere free energy is given by

$$\Delta G_{c,outer} = \Delta H_{c,outer} - T\Delta S_{c,outer} \qquad (26)$$

The surface potential drop, ψ_o, characterizes the entropic contribution to $\Delta G^o_{c,outer}$, and is given by the Grahame relations, which for a planar interface is

$$\left(\frac{\partial\psi}{\partial\boldsymbol{n}}\right)_o = \psi_o = \frac{kT}{e} arccosh\left[\frac{ze\sigma^2}{4\varepsilon\varepsilon_o kTC} + 1\right] \qquad (27)$$

and for cylindrical curved surfaces is defined by

$$\frac{e}{kT}\frac{4\pi\sigma}{\kappa} = 2sinh\left(\frac{\psi_o}{2}\right)\sqrt{1 + \frac{\frac{K_1^2(A)}{K_0^2(A)} - 1}{cosh^2\left(\frac{\psi_o}{4}\right)}} \qquad (28)$$

The enthalpic contribution is considered negligible but can be determined from commonly available solution activity models such as MINTEQ and PHREEQC.

3.4. Inner sphere

Analogously, the inner sphere free energy is given by free energy change of both electrolyte and water

$$\Delta G_{c,inner} = \Delta H_{c,inner} + e\psi_s \tag{29}$$

The entropic contribution to the electrolyte Stern free energy is determined by the inner sphere (Stern) potential, ψ_s, which has contributions from charges on both sides of a layer and within it. The potential drop across the Stern layer one side of a layer, ψ_{S1}, is modeled with the Stern layer capacitance

$$\psi_{S1} = \frac{\sigma_o d_s}{\varepsilon \varepsilon_o} \tag{30}$$

where d_s is the thickness of the Stern layer and σ_o is the structural charge of the layer. In general, binding sites are occupied by either water or cations. Water in Stern layer sites is replaced with ions as α decreases. When $\alpha = 1$ there are no cations in the Stern layer and the drop in Stern potential is

$$\psi_{S1,H_2O} = \frac{\sigma_o d_{H_2O}}{\varepsilon \varepsilon_o} \tag{31}$$

For ions, $d_s = 2r$, where r is the cation radius, and ε depends on the ion type. The dielectric constant of an ion can be related to the ionic polarizability via the ionic susceptibility, χ, which is the polarizability per unit volume. This gives

$$\chi = \varepsilon - 1 \tag{32}$$

The Stern potential for ionic sites is therefore given by

$$\psi_{S1,ion} = \frac{2\sigma_o r}{(\chi+1)\varepsilon_o} \tag{33}$$

The Stern potential drop on one side of a layer, ψ_{S1}, is modeled as a set of parallel capacitors consisting of either water or ions in ditrigonal cavities whose capacitance is additive

$$\psi_{S1} = \left(\frac{(1-\alpha)}{\psi_{S1,ion}} + \frac{\alpha}{\psi_{S1,H_2O}}\right)^{-1} = \frac{\sigma_o}{\varepsilon_o}\left(\frac{(1-\alpha)(\chi+1)}{2r} + \frac{\alpha \varepsilon_{H_2O}}{d_{H_2O}}\right)^{-1} \tag{34}$$

Equation (34) gives the Stern potential contribution from ions on one side of a layer only, but inner-sphere complexation also affects cations on the opposite side. A layer itself acts as a capacitor such that inner-sphere complexation in the Stern layer on the second side of a layer creates a net potential of

$$\psi_2 \cong -\frac{\sigma_o d_{layer}}{\varepsilon_{layer}\varepsilon_o} \tag{35}$$

neglecting the contribution from the outer sphere ions on side 2. The total Stern layer potential on side one is the parallel combination of ψ_1 from side one and ψ_2 from side two, which is

$$\psi_S = \left(\frac{(1-\alpha)}{\psi_{S1}} + \frac{(1-\alpha)}{\psi_{S2}}\right)^{-1} = \left(\frac{(1-\alpha)\varepsilon_o\left(\frac{(1-\alpha)(\chi+1)}{2r} + \frac{\alpha\varepsilon_{H_2O}}{d_{H_2O}}\right)}{\sigma_o} - \frac{(1-\alpha)\varepsilon_o d_{layer}}{\sigma_o}\right)^{-1} \quad (36)$$

$$\psi_S = \frac{\sigma_o}{(1-\alpha)\varepsilon_o}\left(\frac{(1-\alpha)(\chi+1)}{2r} + \frac{\alpha\varepsilon_{H_2O}}{d_{H_2O}} - \frac{\varepsilon_{layer}}{t}\right)^{-1} \quad (37)$$

The change in cation enthalpy at the interface due to bending is expected to be negligible for low curvature, *H*, especially when the Debye screening length is smaller than a lattice constant. Large curvature may give rise to non-linear combinations of parallel and series capacitance between charge sites. However, we expect the most significant enthalpic effect to be the dehydration of cations. Values for the hydration free energy, ΔG_h have been tabulated for the monovalent cations(*7*) and can be shown to reproduce the observed behavior of cations at Mt interfaces (Fig. 1i).

3.5. Mixing free energy

The entropic contribution to the mixing energy is given by Eq. (20). The enthalpic contribution to the mixing energy is due to the change in dielectric constant of the inner and outer-sphere configurations as a function of *α*, which does not change the qualitative behavior of ion complexation over a reasonable range of values.

3.6. Complexation free energy

Combining Equations (20), (22), (24), (29), and (37) gives the total complexation free energy

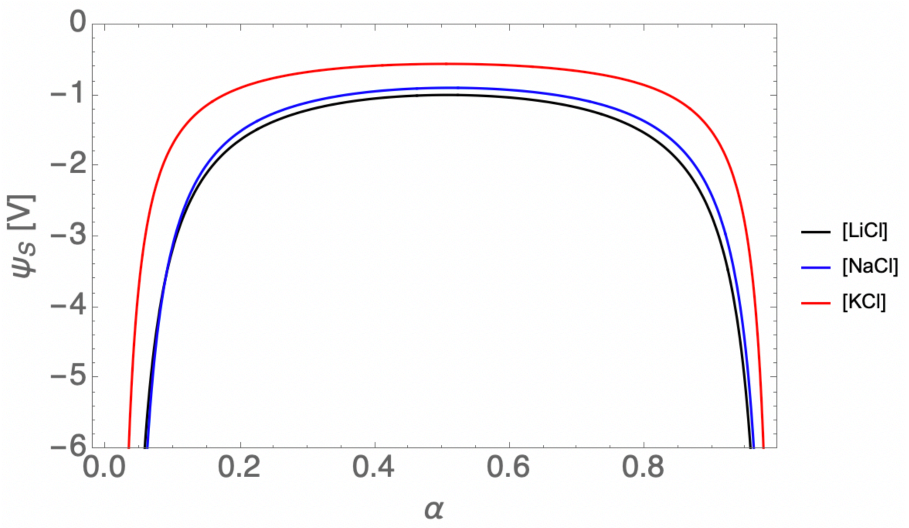

Figure S15. Stern layer potential for Li, Na, and K at Mt surface as a function of *α*.

$$\Delta G_h = -kTln\left(\frac{1-\alpha}{\alpha}\frac{a_{H_2O}}{[X^+{}_{(aq)}]}\right) - e(\psi_o + \psi_s) - \Delta G_{c,bend} \tag{38}$$

The graphical solution to Equation (38) for LiCl is shown in Fig. S15, which quantitatively predicts the transition between nematic and isotropic structures above electrolyte concentrations of approximately 10^{-4} M observed in Fig. S2, under the assumption that the layers are planar (i.e., no curvature energy). The introduction of curvature allows stable binding configurations to exist at elevated electrolyte concentrations because it shifts the right-hand side of Equation (38) along the vertical (energy) axis. However, because curvature can be signed (via Equation (23)) $\Delta G_{c,bend}$ shifts in opposite directions depending on whether the convex or concave side of a layer is being considered, bending can have an asymmetric effect on binding configurations.

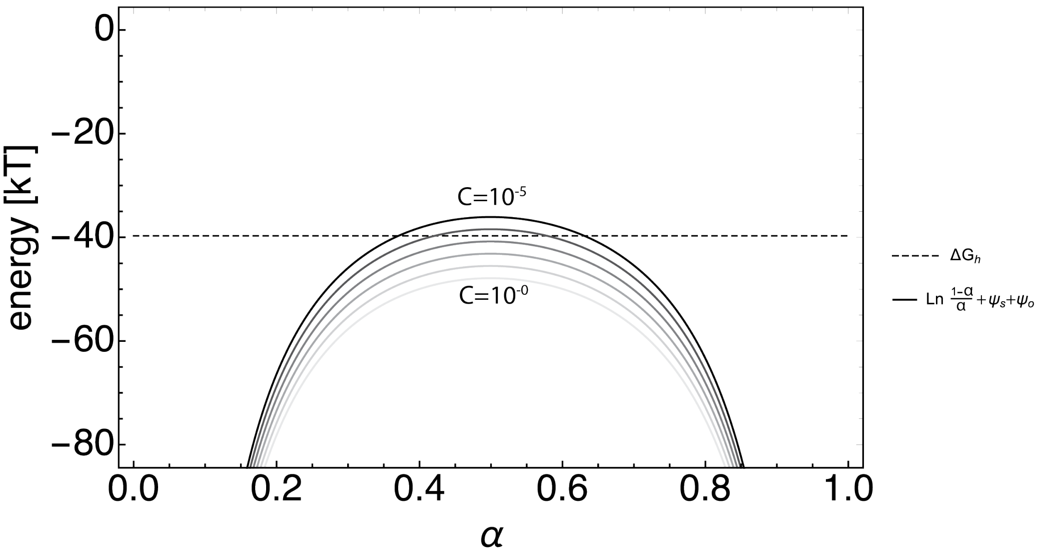

Figure S16. Graphical solution to Equation (38) for Li-Mt in LiCl. No stable binding configurations exist for planar layers above approximately $5x10^{-4}$ M, which is consistent with the transition from nematic to curved, isotropic structures observed in Fig. S2.

The value of α that satisfies Equation (38) gives the fraction of outer-sphere counterions as a function of the water activity, electrolyte concentration, and interfacial potential.

$$\alpha = \frac{1}{1+\frac{[X^+{}_{(aq)}]}{(a_{H_2O})}e^{-\frac{\left(e\psi_O(\alpha)+e\psi_S(\alpha)-\Delta G_{c,bend}-\Delta G_h\right)}{kT}}} \tag{39}$$

Equation (39) conveys the extent to which complexation and curvature are coupled to the bulk solution composition, i.e., the emergence of complexation waves. As shown in Figure S14, the value of α can change vary between 0 and 1 depending on the water activity and electrolyte concentration. As the electrolyte concentrations differ on either side of a curved layer, the fraction of unbound cations changes. The effects of changing α on a given layer affect ψ_o and ψ_s, which propagate to other layers.

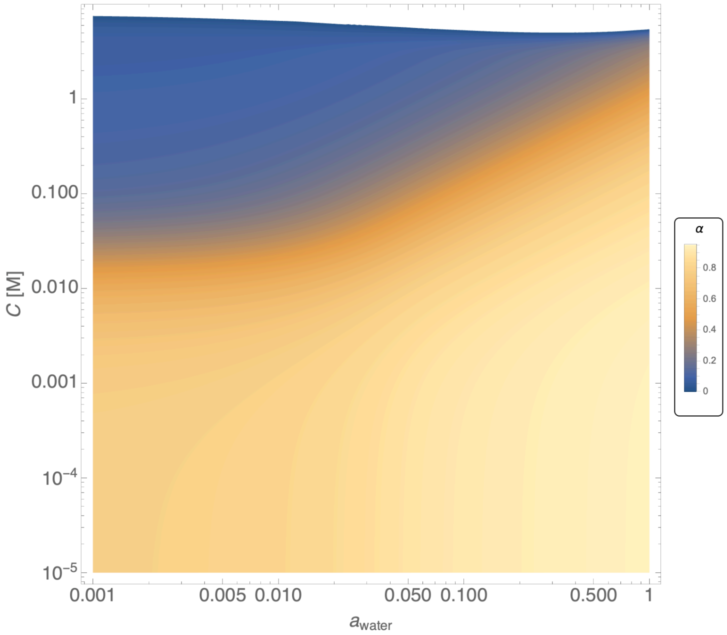

Figure S17. Fraction of bound counterions as a function of water activity and electrolyte concentration in LiCl. The transition between mostly unbound and mostly bound occurs in the concentration range between 0.01 – 1 M and depends strongly on the water activity.

Curvature alters the binding configurations available to the system by shifting the right-hand side of Equation (38) vertically (i.e., along the energy axis). This enables stable binding configurations that are not stable for planar layers (Fig. S16). However, because the sign of the curvature can influence the total bending energy, Equation (23) indicates that the shift of the right-hand side of Equation (38) can go in opposite directions on opposite sides of a layer.

Differences between Li, Na, and K can be seen in the solution to Equation (38) at equivalent electrolyte concentration, reflecting the experimental conditions described in Fig. 1i. Under the assumption of planar layers, plots of Equation (38) at 1 M electrolyte do not have any stable states. However, the energy difference between the right- and left-hand sides, corresponding to the curvature energy needed to stabilize ion complexation at a given value of α, decreases rapidly going from Li to Na to K. This is consistent with the trend observed for the diffusivities of Mt layers in the respective electrolyte solutions, with lower curvature energy corresponding to less charged layers ($\alpha = 0.5$) that interact weakly.

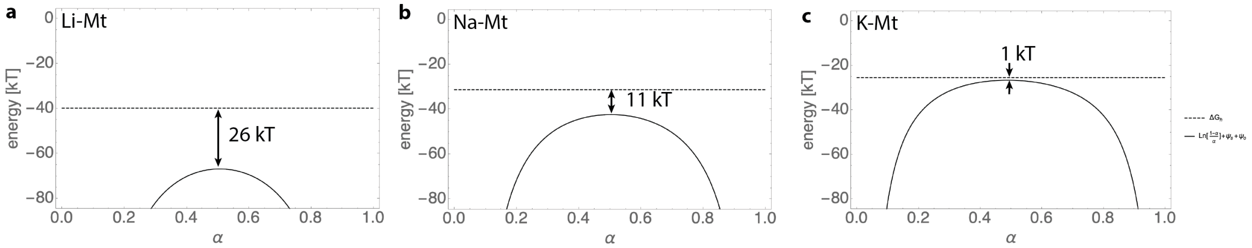

Figure S18. Graphical solution to Equation (38) for (a) Li-Mt, (b) Na-Mt, and (c) K-Mt.

Relating the result of Equation (39) to the experimental layer or tactoid diffusivities requires understanding the effect of changing the interfacial charge, via α, on the interfacial potential, ψ, which causes layers to repel. It can be shown(*4*) that the interaction energy, W, between two charged interfaces in a screened electrolyte is

$$W = 128C\kappa^{-1} tanh\left[\frac{ze\psi_o}{4kT}\right]^2 e^{-\kappa D} \tag{40}$$

In the limit that the electrolyte concentration is high, ψ_o is on the order of $4kT$ and $W \propto \psi_o e^{-\kappa D}$. This is an expression of the Debye-Huckel approximation for the potential at a distance *D* from the interface

$$\psi(D) = \psi_o e^{-\kappa D} \tag{41}$$

Which can be rewritten by substituting Equation (27) into Equation (41)

$$\psi(D) = \frac{kT}{e} arccosh\left[\frac{ze(\alpha\sigma)^2}{4\varepsilon\varepsilon_o kTC} + 1\right] e^{-\kappa D} \tag{42}$$

Because arccosh(x^2+1) ~ x at high electrolyte concentrations,

$$\psi(D) \propto \alpha e^{-\kappa D} \tag{43}$$

Thus, the repulsive strength between layers is directly proportional to the surface charge, which is proportional to the exponential of the hydration energy as given in Equation (39) for a constant value of α and constant curvature,

$$W \propto \frac{[X^+{}_{(aq)}]}{(a_{H_2O})} e^{-\frac{\Delta G_h}{kT}} e^{-\kappa D} \tag{44}$$

To summarize, the average diffusivity of layers and/or tactoids in a screened electrolyte is established by the strength of the interactions between interfaces as they approach, which is proportional to the surface charge and therefore the exponential of the ion hydration energy. However, this is only the *average* diffusivity, as the stochastic fluctuations in layer diffusivity are modulated by the propagation of complexation waves, which alter the surface charge and the bending component of the surface potential to give rise to avalanche transitions between states via asymmetric distributions of electrolyte throughout a collection of interacting layers.

Supplementary References

1. D. M. Moore, J. R. C. Reynolds, *Xray diffraction and the identificaiton and analysis of clay minerals*. (Oxford University Press, New York, New York, ed. 2, 1997).
2. D. Ren, C. Ophus, M. Chen, L. Waller, A multiple scattering algorithm for three dimensional phase contrast atomic electron microscopy. *Ultramicroscopy* **208**, (2020).
3. E. Paineau *et al.*, Aqueous suspensions of natural swelling clay minerals. 1. Structure and electrostatic interactions. *Langmuir* **27**, 5562-5573 (2011).
4. J. Israelachvili, Intermolecular and Surface Forces. *Academic Press*, (2011).
5. K. Norrish, The Swelling of Montmorillonite. 120-134 (1954).
6. B. Duplantier, R. E. Goldstein, V. Romero-Rochin, A. I. Pesci, Geometrical and Topological Aspects of Electric Double Layers near Curved Surfaces. *Physical Review Letters* **65**, 508-511 (1990).
7. G. Lamoureux, B. Roux, Absolute Hydration Free Energy Scale for Alkali and Halide Ions Established from Simulations with a Polarizable Force Field. *Journal of Physical Chemistry B* **110**, 3308-3322 (2006).
